# Neurological complications of left atrial myxoma: a case report on stroke with left atrial myxoma and postoperative brain metastasis and cerebral aneurysm

**DOI:** 10.3389/fnimg.2024.1524901

**Published:** 2024-12-19

**Authors:** Xudong Ai, Qingqing Shao, Xueyan Tian, Yicheng Zhou, Tiantian Zhou

**Affiliations:** ^1^Department of Radiology, Tongji Hospital, Tongji Medical College, Huazhong University of Science and Technology, Wuhan, China; ^2^Computed Tomgraphy Department of the Fifth Affiliated Hospital of Dali University, Baoshan, China; ^3^Department of Medical Imaging, Wuhan Pulmonary Hospital, Wuhan, China

**Keywords:** left atrial myxoma, tumor resection, ischemic stroke, brain metastasis, aneurysm, radiological imaging

## Abstract

Atrial myxoma is a rare benign tumor that can cause a variety of complications, including cerebral infarction. We present a case of a 52-year-old female patient who developed cerebral infarction caused by an atrial myxoma. The patient underwent successful surgical resection of the tumor, and the infarction was managed accordingly. However, 15-months post-surgery, the patient developed new neurological symptoms. Imaging studies revealed multiple cerebral metastases, consistent with the possibility of seeding of tumor cells. This rare complication emphasizes the importance of long-term monitoring after the resection of atrial myxomas. The occurrence of metastasis in the brain, though rare, should be considered in follow-up care, particularly in patients who have had embolic events related to atrial myxomas. Our case highlights the potential for cerebral myxoma metastasis even after initial successful surgical intervention, underscoring the need for comprehensive follow-up and vigilant monitoring of such patients.

## Introduction

Atrial myxoma is a rare benign primary cardiac tumor (Jaravaza et al., 2021), most commonly arising from the endocardial surface of the atria (Reynen, 1995). While it is generally considered non-malignant, a small subset of cases has shown potential for recurrence and even metastatic spread. Tumor fragmentation poses a significant risk, as embolization of detached fragments can result in systemic embolic events, and, in rare instances, lead to tumor metastasis (Hau et al., 2020; Alrohimi et al., 2019; Waliszewska-Prosół et al., 2018).

We present a case involving a 52-year-old female patient who was initially admitted with chest pain, subsequently developing an ischemic stroke that resulted in left-sided hemiparesis. Imaging studies revealed the presence of an atrial myxoma, which was complicated by the acute ischemic event. The patient underwent thoracotomy for the resection of the tumor. However, 1-year post-surgery, multiple brain metastases were detected. Herein, we present a rare case of tumor metastasis after the resection of atrial myxoma. And we recommend closely monitoring MRI follow-up after cerebral infarction caused by atrial myxoma, based on a comprehensive literature review and our experience with this case of multiple cerebral myxoma metastases.

## Case presentation

**Figure 1:**
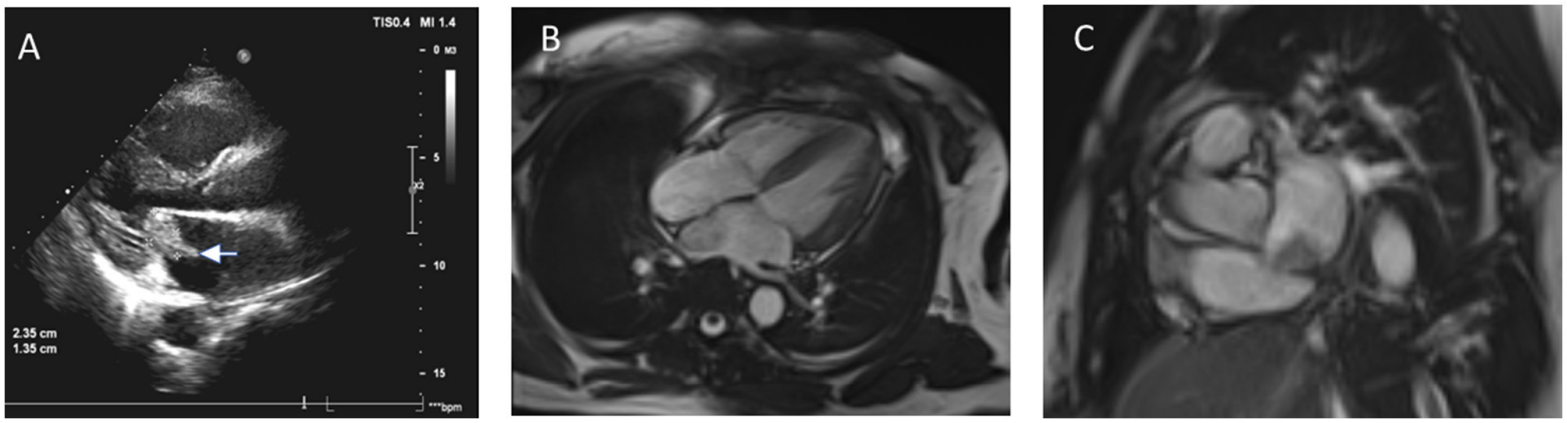
**(A)** Echocardiogram shows a 2.3 × 1.3 cm hyperechoic mass in the left atrium, attached to the interatrial septum. **(B, C)** MRI images reveal an irregular mass in the left atrium (~3.9 × 2.6 cm) with iso-intense signal on T1-weighted and hyperintense signal on T2-weighted images, attached to the interatrial septum with visible mobility.

A 52-year-old female patient was admitted with symptoms of palpitations, chest pain, and weakness in her left lower limb with no prior medical history of conditions such as hypertension, diabetes, atrial fibrillation, smoking, or any other relevant factors. Emergency echocardiography revealed an abnormal echogenic mass in the left atrium (Figure 1A), suspected to be a thrombus or of other possible etiologies, and left atrial enlargement. Cardiac MRI identified an irregular mass within the left atrium, with a maximum cross-sectional diameter of ~3.9 × 2.6 cm (Figures 1B, C). This mass was attached to the interatrial septum and demonstrated good mobility.

**Figure 2:**
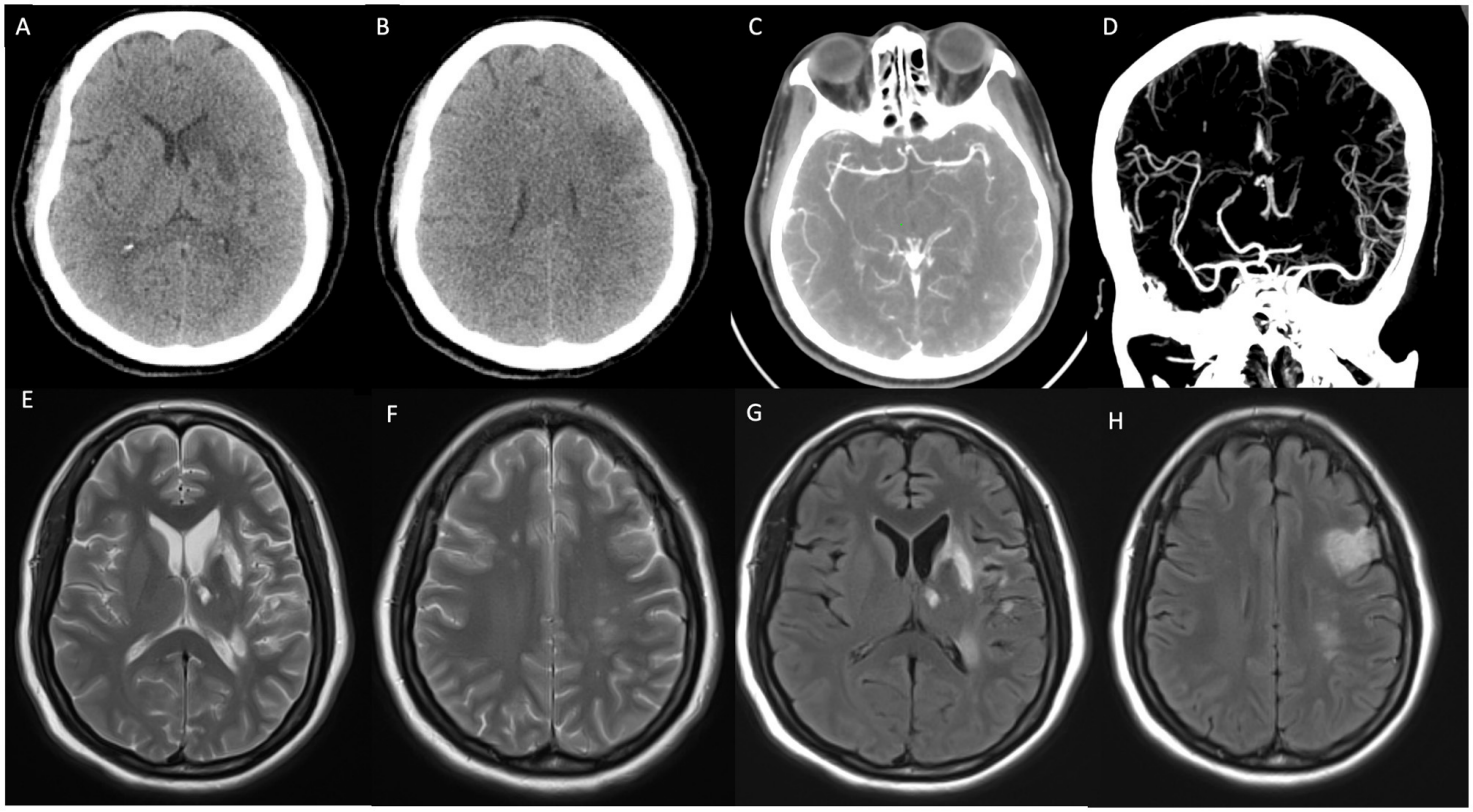
**(A, B)** Non-enhanced CT (NECT) indicates ischemic infarction in the deep region of the left temporal lobe. **(C, D)** CT angiography (CTA) shows localized stenosis in the M2 segment of the left middle cerebral artery. **(E–H)** T2-weighted imaging (T2WI) and T2 FLAIR demonstrate patchy hyperintense signals in the bilateral frontal lobes, left basal ganglia, thalamus, parietal-temporal lobes, and hippocampus.

Concurrently, the patient exhibited left-sided limb weakness. CT scans detected hypodense lesions in the left frontal lobe, basal ganglia, and insula, indicating potential cerebral infarction (Figures 2A, B). CTA showed localized stenosis in the M1 segment of the left middle cerebral artery (Figures 2C, D). Brain MRI revealed abnormal signals in multiple brain regions, suggesting infarction foci (Figures 2E–H).

The initial diagnoses included acute coronary syndrome with non-ST-elevation myocardial infarction, left atrial myxoma, and cerebral infarction. Once the patient's cerebral infarction symptoms stabilized, she underwent left atrial myxoma resection under general anesthesia and cardiopulmonary bypass. The tumor, which was gelatinous and partially ruptured with bleeding, measured 4 × 4 cm and was completely excised along its pedicle.

**Figure 3:**
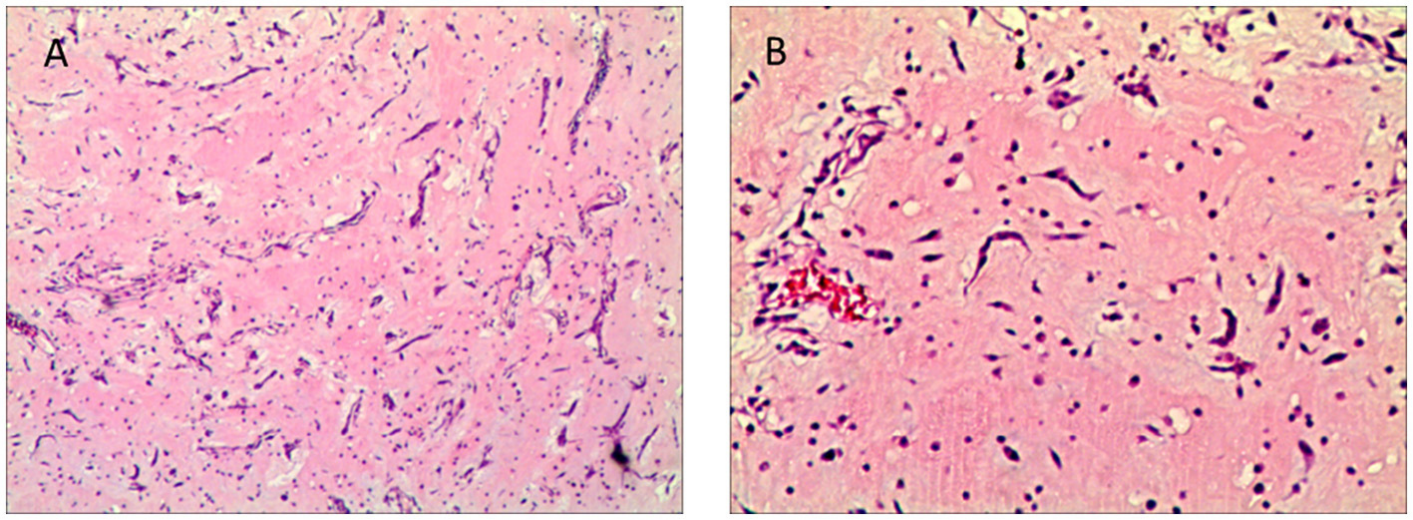
**(A, B)** Histopathological image of cardiac myxoma (H&E stain, 40 ×, 100×). The image shows cardiac myxoma tissue with round or polygonal tumor cells surrounded by abundant mucinous stroma under hematoxylin and eosin staining at 40×, 100× magnification.

Histopathological analysis confirmed the diagnosis of left atrial myxoma (Figure 3), revealing tumor cells surrounded by abundant mucinous stroma. Postoperatively, the patient received no additional treatment after discharge.

**Figure 4:**
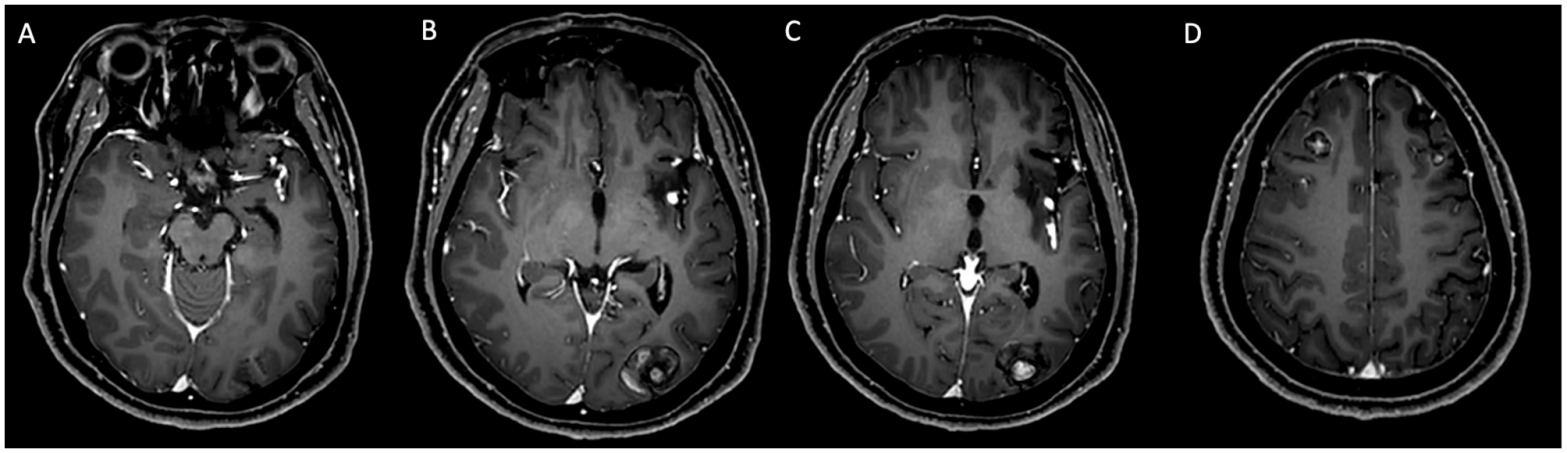
**(A–D)** MRI with contrast enhancement: follow-up examination 15 months post-surgery. Nodular enhancements were observed in the bilateral frontal lobes, right parietal lobe, and left occipital lobe, with the largest lesion measuring 25 × 20 mm in the left occipital lobe. Additionally, non-enhancing areas were noted in the left corona radiata and basal ganglia.

**Figure 5:**
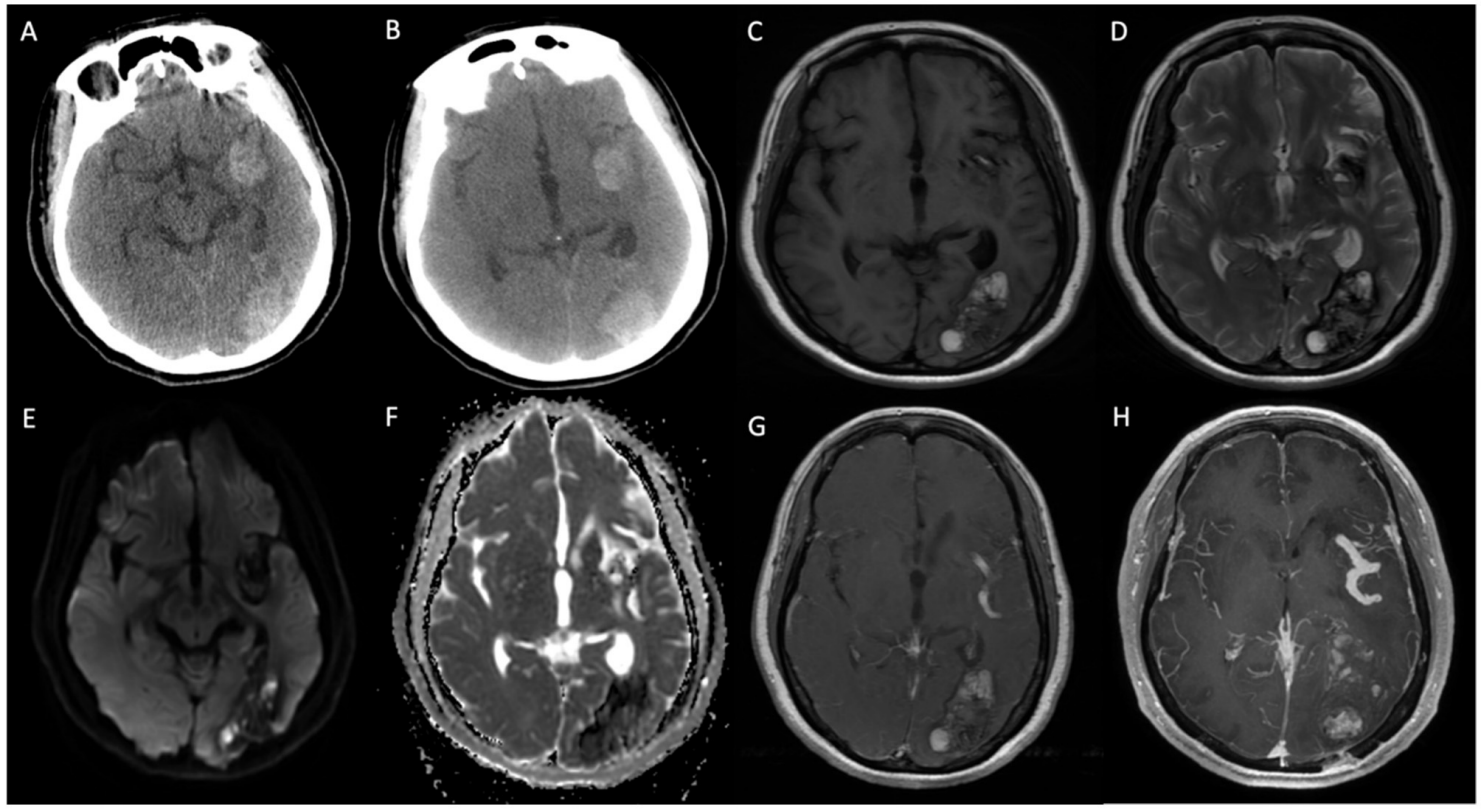
Follow-up examination 23 months post-surgery. [**(A, B)**; CT] Hyperdense masses with hemorrhagic components observed in the left temporal and occipital lobes. [**(C–F)**; MRI] Abnormal signals in the left basal ganglia and occipital lobe on T1- and T2-weighted imaging, with partial diffusion restriction in the left temporo-occipital lobe on DWI/ADC. [**(G, H)**; T1WI + C] Multiple enhancing nodules observed, with aneurysmal dilation in the M1 and M2 segments of the left middle cerebral artery.

Fifteen months later, the patient presented with new neurological symptoms, including headaches, nausea, and vomiting. Follow-up MRI revealed multiple metastatic lesions in multiple brain regions and signs of cerebral malacia in the left corona radiata and basal ganglia (Figure 4, MRI+C). By the 23 months post-surgery, head CT showed an increase in hyperdense lesions across multiple brain regions, indicative of progressive metastatic disease (Figures 5A, B). Abnormal signals in the left basal ganglia and occipital lobe on T1- and T2-weighted imaging, with partial diffusion restriction in the left temporo-occipital lobe on DWI/ADC (Figures 5C–F), and aneurysmal dilation was observed in segments of the left middle cerebral artery (Figures 5G, H), suggesting potential hemorrhagic metastasis from the myxoma.

The increase in lesion number and size supported a diagnosis of recurrent cardiac myxoma with multiple intracranial metastatic sites.

**Figure 6:**
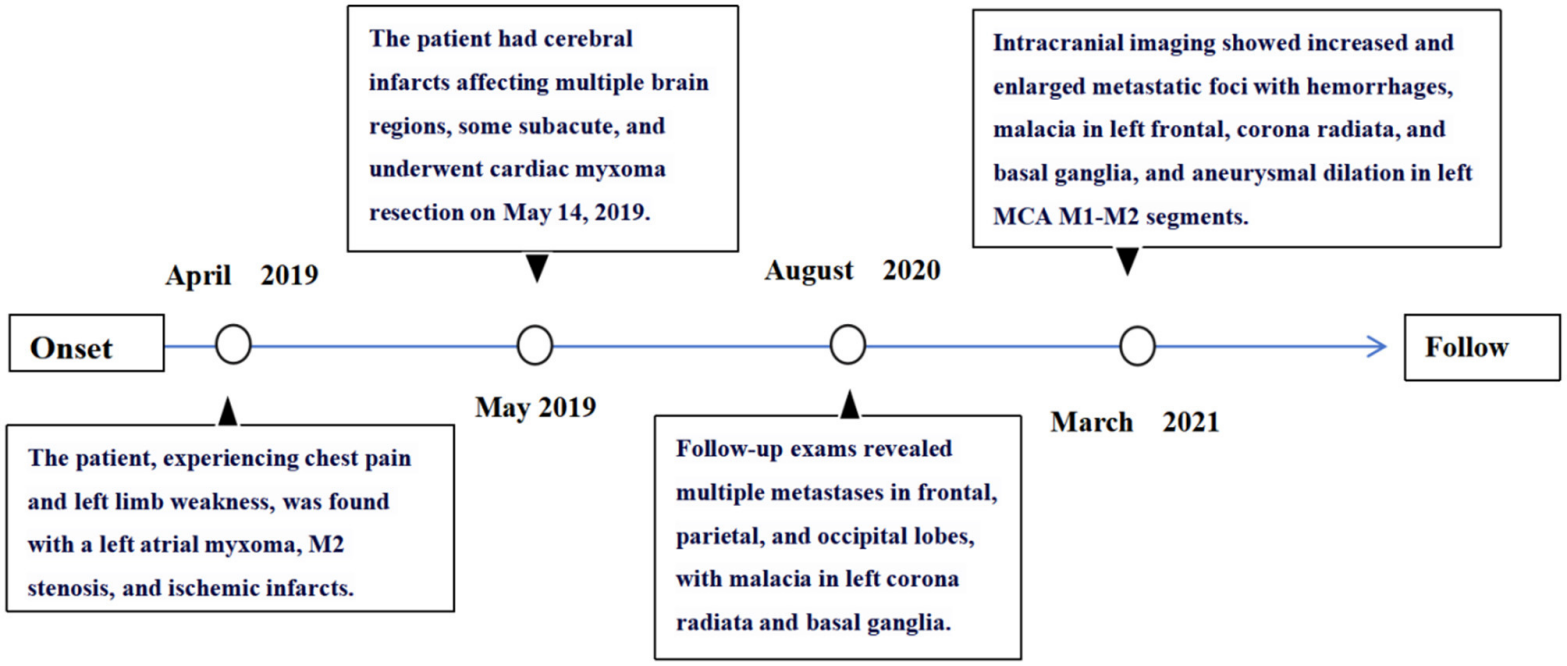
The timeline of the case.

The timeline (Figure 6) summarizes the progression from initial presentation through diagnosis, surgical intervention, and subsequent complications, illustrating the disease course and highlighting the need for long-term surveillance in cases of atrial myxoma.

## Discussion

Primary cardiac tumors are rare (Ma et al., 2022), with half being myxomas mostly in the left atrium (Aggarwal et al., 2007). Most atrial myxomas are sporadic, but some show familial autosomal dominant inheritance like Carney syndrome, involving multiple tumors and endocrine issues (Reynen, 1995). Here, a patient with a left atrial myxoma had an acute stroke, often misdiagnosed, causing delays. After surgery, inadequate monitoring led to rare metastatic tumors in previously infarcted brain tissue.

Current understanding of this condition is limited, with neither comprehensive intracranial evaluations nor long-term follow-up performed. Atrial myxoma complicated by cerebral embolism often presents as an initial symptom, with embolus detachment leading to narrowing of blood vessels at the infarction site, particularly affecting the middle cerebral artery. In this patient, while metastatic foci developed in the initial infarction area, the supplying arteries progressed from stenosis to aneurysmal dilation, accompanied by tumor hemorrhage. Myxomatous aneurysms are particularly rare, and the mechanisms underlying their formation remain unclear (Hau et al., 2020). Two prevailing theories explain their genesis: (1) the vascular injury theory and (2) the tumoral origin theory. The latter is supported by autopsy pathological evidence and corresponds with the later stages of the disease, where metastatic tumors also exhibit hemorrhagic changes.

The literature lacks adequate description of craniocerebral metastasis mechanisms after atrial myxoma resection (Castaño-Leon et al., 2016). This case report emphasizes the need to assess tumor dissemination risk in patients with atrial myxoma and concurrent acute stroke, challenging the typical surgery outcomes. Even after successful resection, cerebral metastasis risk persists. It highlights rare clinical features of atrial myxoma with neurological complications and postoperative metastasis. For patients with cardiac myxoma, especially those with intracranial lesions, comprehensive evaluations and long-term follow-up are crucial to prevent cerebral metastasis or cerebrovascular diseases. CNS symptoms in patients with a history of cardiac myxoma may indicate cerebral metastasis or aneurysm, requiring prompt and thorough assessment and management (Castaño-Leon et al., 2016; Wan et al., 2019).

Currently, there are no treatment guidelines for systemic metastasis caused by cardiac myxoma. According to the literature, the cumulative recurrence rate of cardiac myxoma is 13%, and recurrences occur in a younger population (42 vs. 57 years), with the risk of recurrence decreasing after 4 years (Elbardissi et al., 2008). Therefore, regular follow-up within 4 years after surgery is recommended, with examinations conducted every 6 months.

## Conclusion

Cerebral metastases and aneurysms are rare post-surgical complications of cardiac myxoma, more common in middle-aged females. They often involve the middle cerebral artery and present with vascular events, headaches, and seizures, while hemorrhage and aneurysm rupture are uncommon. Long-term observation is recommended for myxomatous aneurysms. Post-surgery follow-up is vital for detecting potential cerebral complications. Patients with cardioembolic infarctions and coexisting myxoma should be vigilant.

## Data Availability

The datasets presented in this study can be found in online repositories. The names of the repository/repositories and accession number(s) can be found in the article/supplementary material.
